# What Shall a Homœopathic Physician Read?

**Published:** 1890-05

**Authors:** Wm. Steinrauf

**Affiliations:** St. Charles, Mo.


### WHAT SHALL A HOMŒOPATHIC PHYSICIAN READ?

WM. STEINRAUF, M. D., ST. CHARLES, MO.

The above is surely a very important question. There are most assuredly many good medical and other scientific works that may be studied with great advantage by a physician believing in the unerring law, *Similia similibus curantur*, but the question of the utmost importance to us is, what must I study in order that I may safely, quickly, and surely cure my patients? This is the question of questions to every true and honest follower of the master.

There is such a multitude of new books continually advertised that it would cost a little fortune every year to buy them. And still a doctor that is so situated in this life that a few dollars more or less are of no moment to him should purchase every medical book published by our school and the best works by the dominant school as well, even if it has no other object than to make comparisons and draw conclusions. The only works by allopathic writers in my library are Da Costa on *Medical Diagnosis* and Flint on the same subject; all others are homœopathic.

What medical works shall a homœopathic physician read? Above all, he should read and study Hahnemann's *Organon*. It should be his constant companion. I have read it through four times within the last five years, and I think a young doctor should read it through once a year during the first ten years of his professional career in order to get a thorough understanding of the law. This is what Dr. Lippe once told me he had done. Next to the *Organon*, the *Chronic Diseases* and the *Materia Medica Pura* need to be well studied and consulted daily. These three works by the author of Homœopathy should be found in the library of every true homœopathist.

Singular there should be physicians that claim to practice our system, and laugh at these works as being out of date, etc., and still we know of such men. Shame upon such creatures!

Next to Father Hahnemann, the works of a Jahr and Bœnninghausen, a Hartmann and Rueckert are of inestimable value to the student of Homœopathy. Hartmann and Rueckert, as far as we know, have never been translated, but they may be studied by the German scholar with both pleasure and profit.

But, says one, these are all old books, out of date long ago. Well, even if they are old, what of it? Is the Bible not also old? The true homœopathist should study *old* and *new*, but preferably *old*. I well know some will laugh and sneer at this.

But let them laugh. "He who laughs last, laughs best." We must go back to Hahnemann, back to the fathers, back to the fountain to get Homœopathy pure and simple.

After studying these old masters, we must take up the different materia medicas of our time. Here Dunham's *Materia Medica* and his *Science of Therapeutics* demand our special attention. Hering's *Condensed*, Farrington's *Clinical Materia Medica*, and that grand new work of Allen's.

Hand-books of the materia medica must not be forgotten, for they are gems of the first water. Taken all in all the materia medica must be the principal study of the true homœopathist. All other works can only be used as accessory to this very important branch of our divine art.

